# Functional Characterization and Comparison of *Plasmodium falciparum* Proteins as Targets of Transmission-blocking Antibodies*[Fn FN2]

**DOI:** 10.1074/mcp.RA117.000036

**Published:** 2017-10-31

**Authors:** Daria Nikolaeva, Joseph J. Illingworth, Kazutoyo Miura, Daniel G. W. Alanine, Iona J. Brian, Yuanyuan Li, Alex J. Fyfe, Dari F. Da, Anna Cohuet, Carole A. Long, Simon J. Draper, Sumi Biswas

**Affiliations:** ‡ The Jenner Institute, University of Oxford, Oxford UK; § Malaria Immunology Section, Laboratory of Malaria and Vector Research, National Institute of Allergy and Infectious DiseaseNational Institutes of Health, Rockville, Maryland; ¶ Institut de Recherche en Sciences de la Santé, Bobo Dioulasso, Burkina Faso; ‖ Institut de Recherche pour le Développement, Montpellier Cedex, France

**Keywords:** Malaria, infectious disease, immunology, omics, protein design, SMFA, transmission-blocking, vaccines

## Abstract

*Plasmodium falciparum* malaria continues to evade control efforts, utilizing highly specialized sexual-stages to transmit infection between the human host and mosquito vector. In a vaccination model, antibodies directed to sexual-stage antigens, when ingested in the mosquito blood meal, can inhibit parasite growth in the midgut and consequently arrest transmission. Despite multiple datasets for the *Plasmodium* sexual-stage transcriptome and proteome, there have been no rational screens to identify candidate antigens for transmission-blocking vaccine (TBV) development. This study characterizes 12 proteins from across the *P. falciparum* sexual-stages as possible TBV targets. Recombinant proteins are heterologously expressed as full-length ectodomains in a mammalian HEK293 cell system. The proteins recapitulate native parasite epitopes as assessed by indirect fluorescence assay and a proportion exhibits immunoreactivity when tested against sera from individuals living in malaria-endemic Burkina Faso and Mali. Purified IgG generated to the mosquito-stage parasite antigen enolase demonstrates moderate inhibition of parasite development in the mosquito midgut by the *ex vivo* standard membrane feeding assay. The findings support the use of rational screens and comparative functional assessments in identifying proteins of the *P. falciparum* transmission pathway and establishing a robust pre-clinical TBV pipeline.

## Abstract (precis)

The malaria agent *Plasmodium falciparum* continues to evade control efforts. Despite multiple datasets for the *Plasmodium* sexual-stage transcriptome and proteome, there have been no rational screens to identify candidate antigens for transmission-blocking vaccine (TBV) development. We demonstrate the use of rational screens and comparative functional assessments in identifying proteins of the *P. falciparum* transmission pathway and establishing a robust pre-clinical TBV pipeline.

## Graphical Abstract

**Figure:**
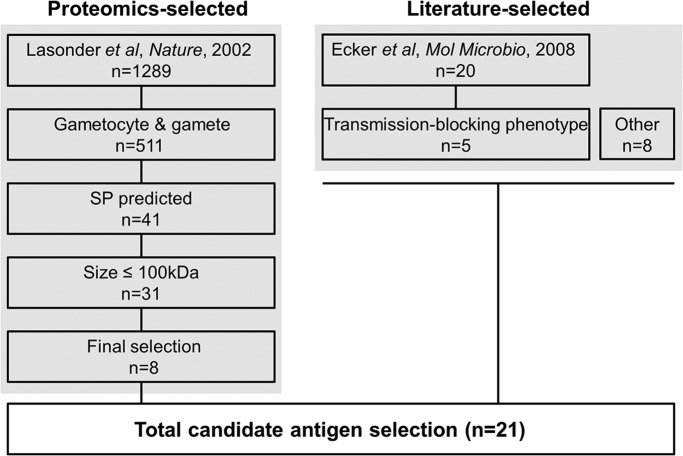

**Highlights**

- Characterization of 12 proteins from across the *P. falciparum* sexual-stages as possible TBV targets.
- Heterologously expressed recombinant proteins recapitulate native parasite epitopes.
- Some recombinant proteins exhibit immunoreactivity when tested against sera from individuals from malaria-endemic Burkina Faso and Mali.
- Purified IgG against the antigen enolase moderately inhibits parasite development in the mosquito midgut.

The global burden of malaria continues to have a profound impact on public health, overwhelming already fragile health care systems and limiting economic stability in the worst affected regions. The etiological agents of human infection include several parasite species of the genus *Plasmodium,* transmitted by the bite of infected female *Anopheles* mosquitos. *Plasmodium falciparum* confers the highest threat of mortality particularly in children under the age of 5 and women with a first pregnancy. Despite public health initiatives reducing the incidence of symptomatic malaria by 30% over the past decade, up to 50% of the world's population remains at risk highlighting the limitations of present interventions (1). Existing gains in malaria control have been largely attributed to preventing transmission between the mosquito vector and human host; these efforts include distribution of long-lasting insecticide-treated bed nets, residual insecticide spraying, and rapid clinical testing coupled with improved drug regimens to lessen the human infectious reservoir. Such strategies have demonstrated success in transitioning high-intensity transmission to low-to-moderate levels across multiple endemic settings (2–4) and renewed the call for vaccines interrupting malaria transmission (VIMTs)^1^ to close the gap in achieving local elimination and global eradication goals (5).

Extensive research efforts have yet to produce a licensed malaria vaccine. The first generation candidate RTS,S/AS01 exhibited only modest efficacy in preventing clinical disease in a Phase III trial (6). Moreover, the lack of transmission-blocking effect in *ex vivo* studies (7) with this vaccine now sets the stage for second-generation formulations to include a VIMT component. VIMTs may target any stage of the malaria parasite lifecycle, but are specifically termed transmission-blocking vaccines (TBVs) when directed against antigens acting on parasite transmission between the human and mosquito (5). These may include parasite sexual-stage proteins as well as components of the mosquito midgut. The transfer of parasites between dramatically different host environments drives a major population bottleneck which is vulnerable to immune recognition (4); mosquito feeding studies such as the standard membrane feeding assay (SMFA) confirm that antibodies to sexual-stage antigens, ingested in the blood meal, can inhibit successful parasite growth in the insect vector. SMFA has historically enabled the screening of monoclonal antibodies to parasite sexual-stage extracts and supported the identification of several TBV candidates. Pfs25, a parasite surface antigen presumed to act in key developmental transitions within the mosquito midgut (8–10), remains the most extensively studied. However, while a Pfs25/Montanide ISA51 formulation showed early potential in a Phase Ia clinical trial, the study was halted due to reactogenicity concerns attributed to the antigen-adjuvant combination (11). So too, the apparent need for high IgG titers (12) called into question whether Pfs25, an antigen with no natural boosting from exposure to human immunity, could elicit antibody responses of a sufficient magnitude and duration for relevance in the field prompting numerous active efforts to improve its immunogenicity.

Identification of new antigenic targets is critical to establishing a robust pre-clinical TBV pipeline. Few parasite antigens have been functionally evaluated for the ability to induce transmission-blocking antibodies, and fewer still have been tested by standardized methods within the same study (13, 14). The majority of targets under active investigation were identified over 30 years ago - broadly grouped into pre-fertilization antigens (gametocyte, gamete) with potential exposure to human immunity, such as Pfs230 and Pfs48/45, and post-fertilization antigens (zygote, ookinete) expressed exclusively in the mosquito vector, such as Pfs25 (15). The sequencing of the *P. falciparum* genome in 2002 identified all possible targets across the sexual-stages (16), and has been followed by numerous global studies of protein expression (17–19). Screens of *Anopheles* proteins acting in the parasite transmission pathway have further generated candidates of interest to TBV development; these are outside of the scope of the present study and have been reviewed extensively elsewhere (20). Still, the field has yet to capitalize on the wealth of knowledge accrued over the past decade. Although rational screening of protein libraries has been applied to both the pre-erythrocytic (21) and erythrocytic stages (22, 23), this approach has never been reported for the identification of new TBV targets.

To determine whether additional proteins may generate transmission-blocking antibodies, we identified 21 candidates from across the *P. falciparum* sexual-stages for heterologous expression and functional characterization. HEK293 cell-based expression of native sequence, full-length ectodomains successfully produced a panel of 12 proteins, which recapitulate parasite epitopes by indirect fluorescence assay (IFA); a proportion exhibit immunoreactivity when tested against sera from individuals living in malaria-endemic Burkina Faso and Mali. Purified IgG generated to the mosquito-stage antigen enolase demonstrates moderate, reproducible inhibition of parasite development in the mosquito midgut.

## MATERIALS AND METHODS

### 

#### 

##### Design of Expression Construct and Protein Synthesis

Sequences corresponding to full-length ectodomains of 22 *P. falciparum* proteins (including Pfs25 as a positive control for the expression construct and platform) were determined by software predicting both signal peptide sequences (24) and membrane-tethering sequences consistent with a transmembrane domain or GPI-anchor (25, 26) (Table I). Synthetic genes were generated based on the *P. falciparum* 3D7 clone (Thermo Fisher Scientific, UK) and codon optimized for expression in *Homo sapiens*. Predicted *N*-linked glycosylation sites were left intact to avoid potentially disruptive modifications of the native sequence (13). The coding region was flanked by unique KpnI and BamHI restriction enzyme sites and subcloned into a modified pENTR4-LPTOS ^TM^ plasmid (23) between a human tPA leader (27) and an affinity sequence encoding a biotin-acceptor peptide (GLNDIFEAQKIEWHE) and hexahistidine tag. Expression plasmids were complexed with polyethylenimine (PEI) and transiently transfected into non-adherent HEK293E cells (28), used for their superior transfection efficiency (data not shown). Soluble recombinant proteins were affinity-purified from whole supernatant using a HisTrap Excel Ni-Sepharose column (GE Healthcare, UK) and subsequently eluted with imidazole. Recombinant proteins were resolved by 4–20% Tris-Glycine gradient sodium dodecyl sulfate polyacrylamide gel electrophoresis (SDS-PAGE) under reducing conditions and visualized by Coomassie brilliant blue R-250 (Bio-Rad, UK). Until use, protein samples were stored in aliquots at −80 °C.

##### Animal Studies and Vaccination

All animal experiments and procedures were performed in accordance with the UK Animals (Scientific Procedures) Act Project License (PPL 30/2889) and approved by the Oxford University Animal Welfare and Ethical Review Body. Six to eight-week-old female BALB/c mice (Harlan, UK), housed in specific-pathogen free environments, were vaccinated with equal amount of vaccines in each leg via the intramuscular route using a protein-in-adjuvant prime-boost regime. Proteins (20 μg total dose per time point), diluted in sterile endotoxin-free PBS, were formulated with the adjuvant prior to vaccinations by mixing a 1:1 volume ratio with 50 μl of Addavax™ (Sigma-Aldrich, UK) per dose. Addavax™ is a squalene-based oil-in-water nano-emulsion with a formulation like MF59® (29). Doses were delivered on days 0, 28, and 56. Vaccine-induced antibody responses were evaluated by small volume tail-bleed on days 27 and 55 and terminal cardiac bleed on day 70. Ovalbumin (OVA) (Invivogen, UK) was delivered by the same schedule and dosage to generate negative control anti-sera.

##### Western Blotting

Standard methods were used to detect proteins by Western blotting (30). After resolution on a 4–20% Tris-Glycine gradient pre-cast gel (Thermo Scientific, UK) by SDS-PAGE and transfer to a nitrocellulose membrane, blots were blocked in 3% BSA/PBS overnight and incubated for 1 h at room temperature (RT) with antibody. Tag-specific detection of the hexahistidine sequence was achieved by anti-pentahistidine monoclonal antibody (Sigma-Aldrich, UK). Antigen-specific detection was performed either with pooled day 70 sera (*n* = 5 BALB/c mice) or 4B7, Pfs25-specific monoclonal antibody (31). Blots were washed with PBS-Tween 20 (PBS/T) for 30 min before incubation with alkaline-phosphatase conjugated goat-anti-mouse antibody for 1 h. Blots were then washed for 30 min with PBS/T, rinsed with deionized water and incubated with SIGMAFAST™ BCIP®/NBT substrate (Sigma-Aldrich, UK) for direct band visualization. Peptide-*N*-Glycosidase F (PNGase F) treatment (New England Biolabs, UK) was carried out in accordance with manufacturer's instructions.

##### Enzyme-Linked Immunosorbent Assay (ELISA)

To detect antigen-specific IgG induced by vaccination, ELISA was performed by standard methods. Briefly, NUNC-ImmunoMaxisorp plates (Thermo Scientific, UK) were coated with 0.1 μg per well of recombinant protein. After blocking with 5% skimmed milk, plates were incubated with diluted test sera (either pre-treated animal sera, or untreated human samples, as described below). Optical density (OD) was read at 405 nm using an ELx800 absorbance microplate reader (Biotek, UK).

For end point titer quantification of IgG generated to the vaccine antigen panel, day 70 sera (*n* = 5 BALB/c mice) were first depleted of antibodies specific to the biotin acceptor peptide and hexahistidine tag; sera were co-incubated with BAP-6HIS peptide (GLNDIFEAQKIEWHEHHHHHH) in 30-fold molar excess of estimated unimmunized mouse immunoglobulin concentration (32) for 30 min at 37 °C. Sera depleted of anti-tag immunoglobulin were then evaluated in duplicate test samples and serially diluted 3-fold. The end point titer was defined as the *x* axis intercept of the dilution curve at an absorbance value three standard deviations (S.D.) greater than the mean OD of pooled sera generated to HEK293E-derived OVA protein (*n* = 5 BALB/c mice). Seroconversion was defined as an achieved end point titer greater than 1.

For antigen immunoreactivity screening, test human sera were diluted to 1:100 and IgG levels were recorded as raw OD. Positive signal was defined as a test sample absorbance greater than 3 S.D. above the mean OD of malaria-naïve adults (UK, *n* = 10) run against the same antigen on the same 96-well NUNC-ImmunoMaxisorp plates (Thermo Scientific, UK). Positive antigens were defined as those antigens with any positive individual serological responders.

##### In vitro *Culture of P. falciparum Gametocytes, Gametes, and Ookinetes*

*P. falciparum* NF54 strain parasites were maintained in *in vitro* blood culture to generate stage V gametocytes (day 16) (33). For gamete preparation, day 16 gametocytes were activated via suspension in standard growth media supplemented with 50% pooled UK naïve serum and incubated at RT for 30 min to promote differentiation to the gamete stage. Gametes were washed three times in RPMI before use. For ookinete preparation, *P. falciparum* NF54 strain gametocyte culture (16–18 days old) was adjusted to 0.15–0.2% stage V gametocytemia at 50% hematocrit. Sixty microliters of pooled human serum was mixed with 200 μl of the gametocyte mixture, and the final mixture was immediately fed to 3–6 day old female *Anopheles stephensi* (Nijmegen strain, *n* = 20) mosquitoes through a membrane feeding apparatus. Mosquito midguts were dissected to harvest the blood meal after 24 h. Pooled blood meals (*n* = 20) containing ookinetes were washed three times in RPMI before use.

##### Indirect Immunofluorescence Assay

Protein localization studies were performed with pooled day 70 sera (*n* = 5 BALB/c mice) by indirect fluorescence assay as previously described (13). Briefly *P. falciparum* NF54 strain gametocytes, gametes, and ookinetes were air dried onto glass slides, and fixed with 4% paraformaldehyde. Gametocytes (intra-erythrocytic) were permeabilized with 0.1% Triton X-100 prior to staining. Slides were blocked for 1 h in blocking buffer (5% BSA in PBS) followed by incubation with test sera (1:100) or control samples diluted in 3% BSA in PBS. Positive controls included Pfs48/45-specific monoclonal antibody 3E12 at 5 μg/ml for the gametocyte and gamete stages and Pfs25-specific monoclonal antibody 4B7 at 5 μg/ml for the gamete and ookinete stages. Anti-OVA sera (1:100) was used as a negative control for all slide sets. Slides were incubated at 37 °C in a wet chamber for 1 h, washed three times in PBS, and then incubated with AlexaFluor 488-conjugated goat anti-mouse IgG (1:2000) for 1 h. Slides were washed five times and mounted with DAPI Vecta-shield (Vector Laboratories, US) and analyzed by fluorescence microscopy on a DMI3000B microscope (Leica Microsystems, Germany).

##### IgG Purification

Total IgG was purified from pooled BALB/c mouse sera as previously described (12). Day 70 mouse sera (*n* = 5 BALB/c mice per test group) were pooled in equal volumes (300 μl serum per mouse) from all mice irrespective of individual antibody titer following protein-in-adjuvant vaccinations. Each protein G-purified total IgG sample was buffer exchanged and adjusted to 3.5 mg/ml in PBS.

##### Standard Membrane Feeding Assay (SMFA)

A qualified methodology for performing SMFA has been previously described (33). Briefly, 60 μl of test sample was mixed with 200 μl of gametocyte culture, consisting of 16–18 day old *P. falciparum* NF54 strain parasites, to a final IgG concentration of 0.75 mg/ml in the feeder; the final mixture was immediately fed to ∼50 3–6 day old female *A. stephensi* (Nijmegen strain) mosquitoes. Purified IgG generated from OVA immunized mice was used as a negative control. Mosquitoes were kept for 8 days and dissected (*n* = 20 per sample) to enumerate the oocysts in the midgut. Only midguts from mosquitoes with any eggs at the time of dissection were analyzed. All SMFA was conducted in the presence of active human complement.

##### Human Serum Collection

Sera samples from Malian adults (*n* = 36) were obtained from a study conducted in accordance with the Ethics Committee of the Faculty of Medicine, Pharmacy and Odontostomatology at the University of Bamako and the Institutional Review Board (IRB) of the National Institute of Allergy and Infectious Diseases (NIAID) (Ref: NCT00669084) (34). Malian serum samples were collected from clinically healthy adults who lived in a malaria endemic area, Kenieroba, in Mali. After obtaining a written informed consent, ∼8 ml of blood per person was collected using a serum separation tube, and the tube was centrifuged 1300 × *g* for 10 min. The resulting serum was aliquoted into small 3–4 cryotubes and kept at −80 C° until used. Sera from Burkina Faso adults (*n* = 32) were collected with approval from the Centre MURAZ Ethical Review Committee (Ref: 002–2014/CE-CM). Burkina Faso serum samples were also collected as above.

UK adult sera (*n* = 10, day 0 samples) were obtained as part of the VAC044 study conducted at the Oxford Vaccine Centre for Clinical Vaccinology and Tropical Medicine, University of Oxford, UK with all necessary approvals granted by the Oxfordshire Research Ethics Committee A (Ref: 11/H0604/2) and the UK Medicines and Healthcare products Regulatory Agency (Ref: 21584/0280/001-0001) (35). US adult sera were purchased from Interstate Blood Bank, Inc. (Memphis, TN).

##### Statistical Analysis

Percent inhibition of mean oocyst intensity (transmission reducing activity, TRA) was calculated by the following formula: 100 × [1 - (mean number of oocysts in the test group)/(mean number of oocysts in the OVA control group)]. The best estimates and the 95% confidence intervals of TRA from a single or multiple feeds were calculated using a zero-inflated negative binomial model as described previously (33).

All statistical tests were performed in R (version 2.15.2) or Prism 5 (GraphPad Software Inc., US). *p* values <0.05 were considered significant.

## RESULTS

### 

#### 

##### Selection of Candidates

**Fig. 1.**
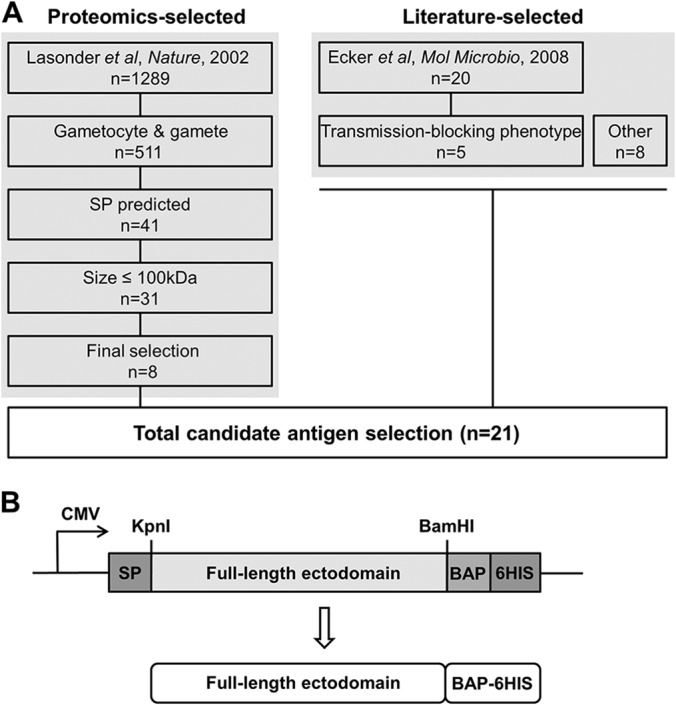
**Design of a panel of *P. falciparum* sexual-stage antigens for expression in the HEK293E cell system.** *A*, Candidate antigens (*n* = 21) were selected from both an unbiased screen of the *P. falciparum* sexual-stage proteome (*n* = 8) and proteins described to be important to sexual-stage biology (*n* = 13). *B*, Synthetic genes were generated from full-length ectodomains based on the 3D7 sequence and subcloned into an expression plasmid encoding an exogenous human tPA signal peptide (SP) and tag containing both a biotinylatable (BAP) and hexahistidine (6HIS) sequence for affinity purification of soluble protein product from whole HEK293E cell supernatant.

In assembling a panel of recombinant *P. falciparum* proteins with sexual stage expression for immunological and functional studies, we included surface-associated candidates of biological interest from literature review (*n* = 13) (Table I), as well as an unbiased selection from the proteome (*n* = 8) (Table II) with an emphasis on those proteins which have not been systematically evaluated as TBV candidates. Literature-based targets included proteins which have historically generated interest in the field as well as five proteins from a knockout screen (PSOP7, PSOP9, PSOP13, CCP5, ASP) which demonstrated defects in parasite development within the mosquito corresponding to a block in transmission (Table I, Fig. 1*A*) (36). Candidates identified from the literature were mainly post-fertilization targets—partly the reflection of a historical trend toward this stage in TBV development. As such, we emphasized proteins with pre-fertilization stage expression in the proteome-based selection. Review of the *P. falciparum* proteome (17) identified 1289 unique proteins by high accuracy mass spectroscopy between the asexual blood stages, gametocytes and gametes. Of these, 511 are expressed in both the gametocyte and gamete stage, though notably most (*n* = 348) are not exclusive to these stages. Forty-one of the 511 are predicted to have a signal peptide. Excluding proteins of a size that may preclude successful heterologous expression (>100kDa) resulted in 31 candidates (supplemental Table S1), including well-characterized stage-specific proteins Pfs16, Pfs25, and Pfs48/45. We chose 5 named and 3 hypothetical targets, which have never been evaluated as possible TBV targets, at random (Table II, Fig. 1*A*). HSP70–2, PDI-8, and PDI-11 are predicted by Wass *et al.* to localize to the *P. berghei* ookinete surface, suggesting biological function in multiple stages (19). The deliberate choice of only 8 candidates was limited by the resources involved in protein expression and downstream animal vaccination, with the intention to expand the study if any of these demonstrate transmission-reducing activity.

**Table I:** Selected candidates from literature review

Name	Protein class	Accession	Size	SP	TMD	N-Glyc	Expression	References	
PSOP7	Putative secreted ookinete protein	PF3D7_1340000	72.2	+	1	7	–	Ecker et al., 2008	(36)
PSOP9^a^	Putative secreted ookinete protein	PF3D7_0828800	85.3	+	2	5	+	Arumugam et al., 2011; Ecker et al., 2008	(36, 54)
PSOP13	Putative secreted ookinete protein	PF3D7_0518800	23.5	+	0	1	+	Ecker et al., 2008	(36)
ASP^b^	Aspartyl protease	PF3D7_0311700	49.4	+	1	1	–	Ecker et al., 2008	(36)
CCP5^c^	Lectin adhesive protein	PF3D7_1451600	100	+	0	8	–	Ecker et al., 2008; Raine et al., 2007	(36, 56)
Pfs47	6-cysteine domain	PF3D7_1346800	50.8	+	2	2	+	Tachibana et al., 2015; van Schajik et al., 2006	(49, 57)
PPLP3^d^	*Plasmodium* perforin-like protein	PF3D7_0923300	93.3	+	1	8	–	Kadato et al., 2004; Kaiser et al., 2004	(58, 59)
PPLP5	*Plasmodium* perforin-like protein	PF3D7_0819200	79.4	+	1	4	–	Kaiser et al., 2004	(36, 59)
SOAP	Secreted ookinete adhesive protein	PF3D7_1404300	22.7	+	0	6	+	Dessens et al., 2003; Nacer et al., 2008	(51, 60)
WARP	von Willebrand factor A domain related protein	PF3D7_0801300	32.0	+	0	1	+	Abraham et al., 2004; Li et al., 2004; Yuda et al., 2001	(52, 53, 61)
Chitinase		PF3D7_1252200	42.8	+	0	3	Low	Isaacs et al., 2012; Langer et al., 2002	(62, 63)
Enolase		PF3D7_1015900	48.7	–	0	4	+	Ghosh et al., 2011	(55)
HYP4	Hypothetical	PF3D7_0417000	32.9	–	1	3	–	Personal communication	

Size is reported in kiloDaltons. Low expression denotes a yield below 300 μg. Abbreviations: SP: presence or absence of signal peptide; TMD: number of predicted transmembrane domains; N-Glyc: number of N-glycosylation site.

^a^PSOP9 is also known as GAMA, or GPI-anchored micronemal antigen.

^b^ASP is also known as plasmepsin IV.

^c^Referred to as CCPs in the *P. falciparum* literature, *P. berghei* homologs are called LAPs (LLCL-lectin adhesive-like proteins).

^d^PPLP3 is also referred to as MAOP, or membrane attack ookinete protein.

**Table II:** Selected candidates from unbiased proteomics review

Name	Protein class	Accession	Size	SP	TMD	N-Glyc	Expression	References	
HSP70–2	Heat shock protein	PF3D7_0917900	72.4	+	0	2	+		
PDI-8	Putative disulfide isomerase precursor	PF3D7_0827900	55.5	+	1	0	+		
GEST	Gamete egress and sporozoite traversal	PF3D7_1449000	28.8	+	0	0	Low	Talman et al., 2011	(64)
PDI-11	Putative disulfide isomerase precursor	PF3D7_1134100	49.2	+	0	4	+		
ICP	Inhibitor of cysteine proteases	PF3D7_0911900	47.0	+	0	8	+	Rennenberg et al., 2010	(65)
HYP1	Hypothetical	PF3D7_0925900	24.7	+	0	3	+		
HYP2	Hypothetical	PF3D7_0303900	23.2	+	0	2	+		
HYP3	Hypothetical	PF3D7_0912400	52.7	+	1	1	–		

Size is reported in kiloDaltons. Low expression denotes a yield below 300 μg. Abbreviations: SP: presence or absence of signal peptide; TMD: number of predicted transmembrane domains; N-Glyc: number of N-glycosylation sites.

The primary study objective was to develop a panel of recombinant proteins with proven expression in the sexual stage and to characterize these proteins as biological targets of transmission-blocking antibodies. As such, we did not exclude proteins that may not ultimately be competitive vaccine antigens, for example electing to express enolase and HSP70–2 for biological interest despite speculative surface expression and the existence of human homologs.

##### Generation of a Panel of Sexual-stage P. falciparum Proteins

Synthetic genes were generated based on the *P. falciparum* 3D7 strain sequence, spanning the coding region after the parasite signal peptide up to the first transmembrane domain or GPI-anchor, if present, to support solubility of the recombinant product. The native sequence was codon-optimized for expression in *Homo sapiens* and predicted N-glycosylation sites were left intact. The full-length ectodomains—flanked by unique KpnI and NotI restriction enzyme sites—were then subcloned into an expression construct encoding an exogenous N-terminal human tissue plasminogen activator (tPA) signal peptide and C-terminal biotin-acceptor and hexahistidine affinity tag (Fig. 1*B*).

**Fig. 2.**
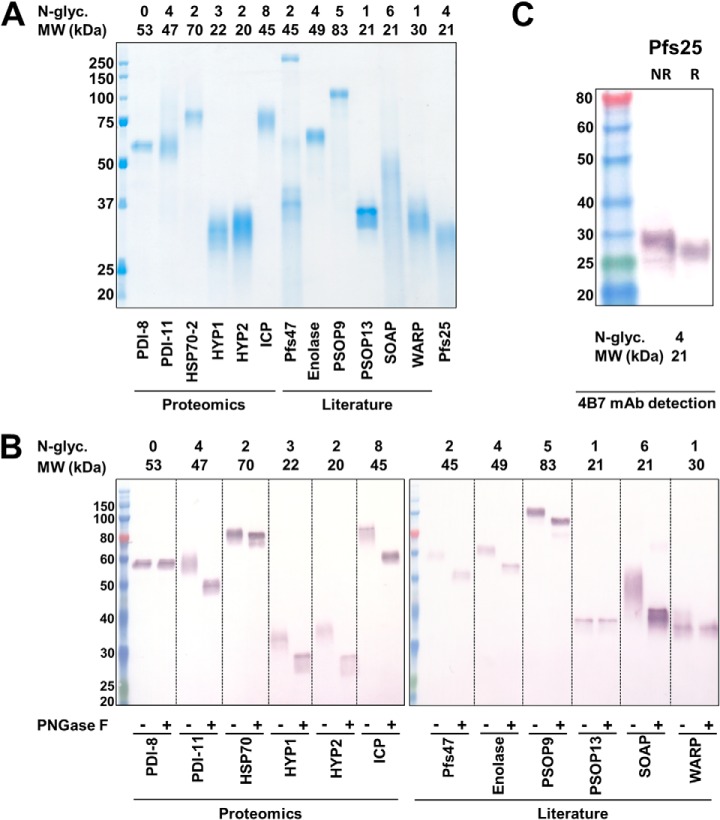
**Heterologous expression of surface and secreted *P. falciparum* sexual-stage proteins.** *A*, Coomassie brilliant blue R-250 staining of recombinant proteins resolved by reducing SDS-PAGE. Each protein has a C-terminal BAP and 6HIS and was purified from whole HEK293E cell supernatant by HisTrap Excel Ni-Sepharose column prior to visualization. *B*, Western blotting detection of hexahistidine tag affinity-purified proteins, which were resolved by reducing SDS-page, blotted, and probed with monoclonal anti-pentahistidine antibody. Each protein is shown with and without treatment by PNGaseF, an amidase which cleaves *N*-linked glycan residues. *C*, Western blotting detection of affinity-purified Pfs25 by 4B7 monoclonal antibody after SDS-PAGE under reducing and non-reducing conditions and nitrocellulose transfer.

All 21 plasmids corresponding to our candidate antigens were successfully generated; an identical strategy was applied to known TBV candidate Pfs25, which was included as a positive control for our expression construct design. Proteins were transiently expressed in non-adherent HEK293E cells and affinity purified from whole supernatant using a HisTrap Excel Ni-Sepharose column. Fifteen out of 22 proteins (68%) showed detectable expression by Western blotting using an anti-petahistidine antibody, which is in keeping with previous reports for *P. falciparum* protein expression in HEK293E cells (22). Thirteen out of those 15 proteins (87%), including Pfs25, were purified in sufficient quantities for a small-animal vaccination study, *i.e.* equal to or exceeding 300 μg. Insufficient expression was noted for chitinase and GEST, and no detectable expression was observed for PSOP7, PPLP3, PPLP5, CCP5, ASP, HYP3, and HYP4 (Table I and 2). Successful expression did not correlate with molecular weight, predicted N-glycosylation sites, or other protein parameters including instability index, aliphatic index, or grand average of hydopathicity (data not shown). Affinity purified proteins were visualized by Coomassie staining following reducing SDS-PAGE, with bands at sizes or above the predicted molecular weight, apart from Pfs47, which contained possible co-purification products (Fig. 2*A*). To evaluate whether N-glycosylation variants could explain the diffuse banding and increased size, proteins were treated with PNGaseF and reduced. Specific monoclonal anti-pentahistidine antibody detection by Western blotting following reducing SDS-PAGE showed a decreased molecular weight and more uniform banding for all proteins except PDI-8 and a diminished effect for PSOP13 and WARP; indeed PDI-8 does not contain any predicted N-glycosylation sites and PSOP13 and WARP only have one predicted site each (Fig. 2*B*). Notably the glycosylated Pfs25 protein product was detectable by known transmission-blocking monoclonal antibody 4B7 (31), suggesting that this epitope is intact (Fig. 2*C*).

##### Recombinant P. falciparum Proteins Recreate Native Epitopes

**Fig. 3.**
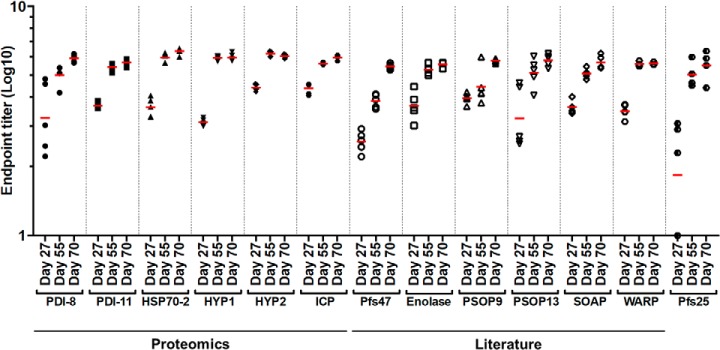
**Generation of antigen-specific IgG to heterologously-expressed *P. falciparum* sexual-stage proteins.** Groups of 5 BALB/c mice were vaccinated intramuscularly with 20 μg of affinity-purified protein in Addavax™ adjuvant on days 0, 28, and 56. Sera were collected at days 27, 55, and 70. For all quantitative immunogenicity studies, sera were depleted of antibodies specific to the biotin acceptor peptide and hexahistidine tag. End point titer ELISA was used to quantify IgG responses to the panel of vaccine antigens. Red bars represent the geometric mean. As seroconversion was defined as an end point titer greater than 1, seronegative mice (Pfs25 group, day 27, *n* = 2) were assigned an end point titer of 1 for analysis and are as such represented on a logarithmic scale. One mouse did not survive to the completion of the study; the Pfs25 group at the day 70 time point represents data from 4 animals.

Anti-sera were generated to the panel of proteins by vaccination of BALB/c mice (*n* = 5 per test group) with 20 μg of affinity-purified protein in Addavax™ adjuvant in three intramuscular doses delivered 4 weeks apart. Sera were collected at days 27, 55, and 70 to reflect antibody titers following the priming dose, first boost, and second boost respectively. Animals seroconverted with the priming dose for all test antigens except the Pfs25 control (Fig. 3); all animals in the Pfs25 group seroconverted by the second vaccination.

**Fig. 4.**
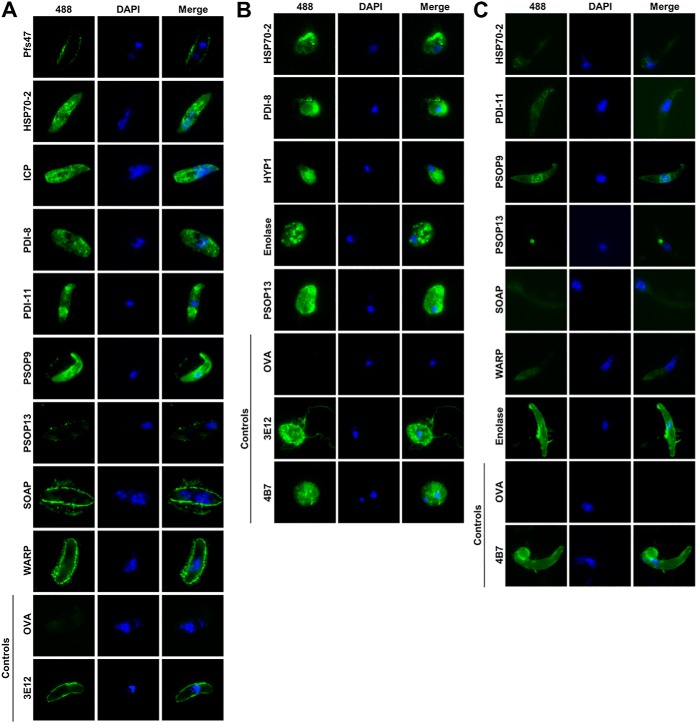
**Recombinant *P. falciparum* sexual-stage antigens recreate native parasite epitopes.** Pooled day 70 anti-sera (*n* = 5 BALB/c mice) generated to the panel of sexual-stage antigens were used to probe parasite epitopes by IFA. IFA identified unique staining patterns of fixed parasite epitopes by pooled anti-sera. *P. falciparum* NF54 strain stage V gametocytes (*A*) and gametes (*B*) were generated *in vitro*. NF54 strain ookinetes were dissected from infected *A. stephensi* mosquito midguts (*C*). Parasites were air-dried onto glass slides, fixed with 4% paraformaldehyde. For gametocyte staining (*A*) the cells were permeabilized with 0.01% Triton X-100 prior to immunostaining. All anti-sera were tested; only pooled anti-sera with detectable reactivity are shown. Stage-specific positive and negative controls were included with each IFA study to calibrate exposure settings; positive controls included Pfs48/54-specific monoclonal antibody 3E12 for the gametocyte and gamete stages and Pfs25-specific monoclonal antibody 4B7 for the gamete and ookinete stages.

To indirectly assess if HEK293E-derived proteins in the sexual-stage panel contain native parasite epitopes, pooled day 70 sera (*n* = 5 BALB/c mice per antigen) specific to each antigen were used to visualize *P. falciparum* NF54 strain parasites. IFA was performed to evaluate serum recognition of gametocytes and gametes generated *in vitro* and ookinetes generated *in vivo* through mosquito blood feeding and dissection (Fig. 4). Nine of 12 sera stained Triton X-100 permeabilized, intra-erythrocytic gametocyte stages (Fig. 4*A*). Five of 12 anti-sera stained non-permeabilized, exoerythrocytic gamete stages (Fig. 4*B*) and 7 of 12 anti-sera stained non-permeabilized ookinetes (Fig. 4*C*). Anti-sera generated to hypothetical protein HYP3 failed to recognize any sexual lifecycle stage by IFA.

##### Recombinant P. falciparum Proteins Are Immunoreactive

**Fig. 5.**
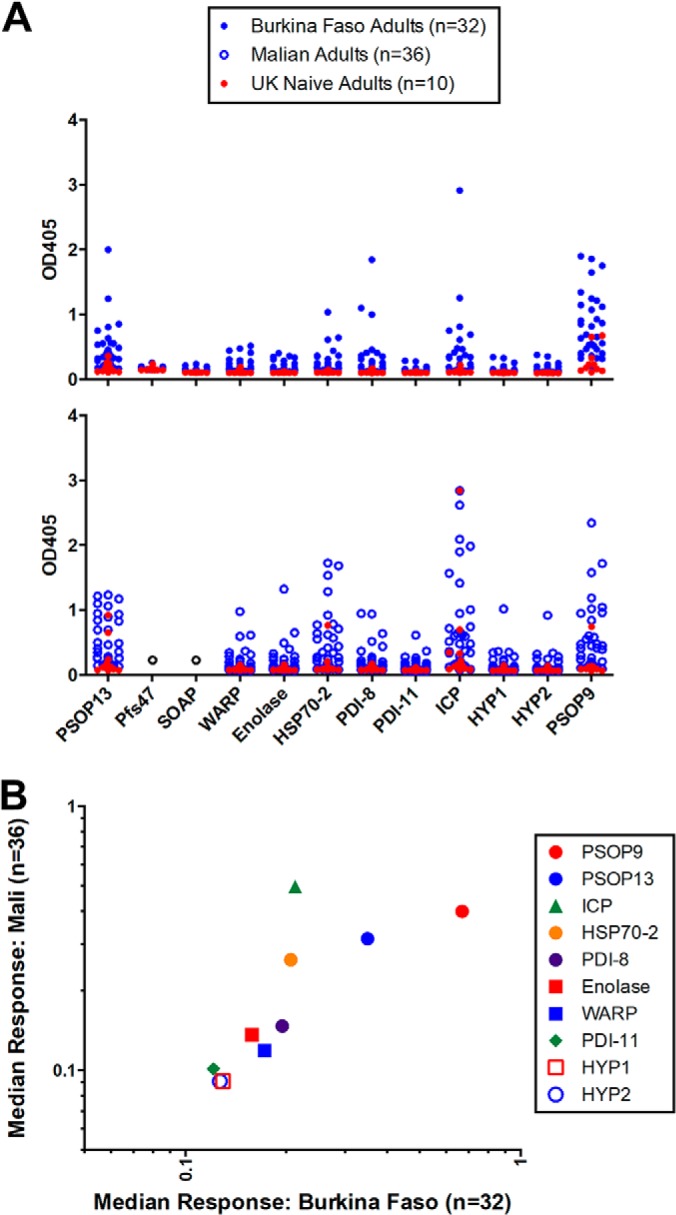
***P. falciparum* sexual-stage recombinant proteins demonstrate immunoreactivity in two independent adult cohorts from malaria-endemic regions.** *A*, ELISA was used to determine the immunoreactivity of heterologously expressed proteins in a cohort of adults from malaria-endemic Burkina Faso (*n* = 36) and Mali (*n* = 32) by quantifying IgG responses, reported as OD at an absorbance of 405 nm. The cutoff for positive response was defined 3 S.D. above the mean optical density of malaria-naïve UK adults (*n* = 10). Limited protein availability precluded testing of Pfs47 and SOAP in the Malian cohort. *B*, The median IgG response to the panel of sexual-stage proteins correlated between the Burkina Faso and Malian cohort with 7 of 12 demonstrating positive immunoreactivity in both cohorts.

To further determine whether the recombinant antigens display native epitopes, we tested immunoreactivity using sera from two cohorts of adults living in malaria-endemic Burkina Faso (*n* = 32) and Mali (*n* = 36) - regions defined by intense and highly seasonal *P. falciparum* transmission (1) - relative to UK adults naïve to malaria infection (*n* = 10). Individuals from Burkina Faso demonstrated positive responses to 7 out of 12 antigens (Fig. 5*A*). The same 7 antigens also demonstrated positive responses among a proportion of Malian samples. In rank order, the highest level of response as defined by median raw OD among individuals from Burkina Faso was to PSOP9, PSOP13, ICP, HSP70–2, and PDI-8, all of which recognized gametocyte-stage parasites by IFA. Immunoreactivity in a cohort of adults in malaria-endemic Mali demonstrated a similar rank order in an independent field site (Fig. 5*B*), suggesting that these recombinant antigens indeed display epitopes seen in native parasite proteins.

##### Antibodies to Enolase Moderately Inhibit Oocyst Intensity by SMFA

**Fig. 6.**
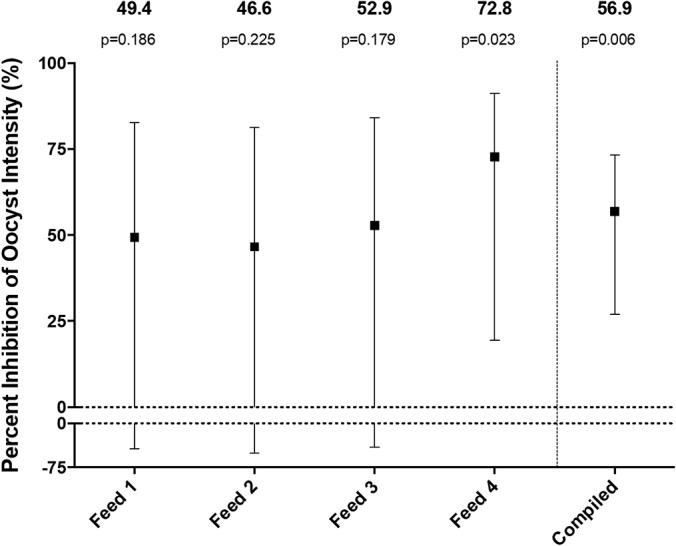
**Enolase elicits significant transmission-blocking activity by SMFA.** Purified IgG from pooled anti-serum generated to recombinant sexual-stage protein enolase was tested by SMFA in the presence of human complement at a final concentration of 0.75 mg/ml in the mosquito blood feeder. Percent inhibition of mean oocyst intensity and 95%CI was evaluated relative to anti-OVA IgG for each of four independent feeding experiments (denoted “Feed 1–4”). To estimate the percent inhibition of oocyst intensity and 95%CI based on these four independent feeding assays, a model was applied which incorporates data from multiple independent feeding experiments to account for assay variability due to feed-to-feed and pint-to-pint differences (33) (denoted “Compiled”).

Sera produced against each recombinant antigen were tested for transmission-blocking antibodies by SMFA. Total IgG was purified from pooled day 70 sera for each protein, and percent inhibition of mean oocyst intensity relative to anti-OVA IgG was calculated for each sample in two independent assays (supplemental Table S2 and S3). Based on the results, the top 3 performing IgG samples (anti-HSP70–2, anti-enolase, and anti-HYP1 IgG) were selected and further evaluated in two additional independent feeds, as SMFA is known to have a lower power to detect low and moderate inhibitions (33). Only anti-enolase IgG demonstrated a reproducible, moderate inhibition of oocyst intensity; TRA in each feed was 49.4, 46.6, 52.9, and 72.8, and the best estimate (95% CI) from 4 feeds was 56.9 (27.0–73.3), which was significant (*p* = 0.006) (Fig. 6). On the other hand, the other two IgG samples did not show consistent inhibition: TRA of anti-HSP70–2 IgG in each assay was −20.0, 98.2, −18.0 and −57.6; TRA was 45.7, 33.8, −10.1 and −28.6 for anti-HYP1 IgG.

## DISCUSSION

The majority of *P. falciparum* malaria endemic geography is defined by low transmission (entomologic inoculation rate < 5) (37), marking a pressing need for innovative technologies such as VIMTs, and specifically TBVs, to target the parasite reservoir in regions working to achieve elimination. We report the use of a mammalian HEK293E platform for the expression of a panel of sexual-stage proteins of enough yield for use in a rodent vaccination study. We have shown that these proteins recapitulate native parasite epitopes as judged by IFA and display immunoreactivity in two independent cohorts from malaria-endemic Burkina Faso and Mali. Moreover, we have evaluated these proteins as vaccine immunogens for the induction of functional, transmission-blocking antibodies in SMFA; this is the first protein library to be screened for functional TRA by SMFA. Anti-enolase IgG demonstrated a significant inhibition of 56.9% based on 4 independent SMFA results.

Progress toward a field-deployable TBV has been hampered by challenges in expressing full-length, high-quality recombinant *P. falciparum* protein libraries in enough quantities for functional screening (38, 39). High-throughput protein expression platforms have been notoriously difficult to establish; the *Plasmodium* genome is A/T rich (over 80%) with abundant repeat regions (16). So too, while the complex tertiary structures and multiple domains of many *Plasmodium* proteins would benefit from a heterologous eukaryotic expression platform, *Plasmodium* cell surface and secreted proteins are, among eukaryotes, uniquely not N-glycosylated because of a lack of appropriate enzymatic machinery (40). Several large-scale attempts have been made in bacterial, yeast, plant, mammalian and cell-free systems, but have been limited by low throughput or the inability to replicate native epitope conformations (reviewed in (15)). Advances in mammalian expression construct and cell line performance (28, 41, 42) have improved success rates in the production of soluble protein for use in immuno-epidemiology (43) and receptor-ligand interaction studies (22, 44). We chose to use transiently transfected, non-adherent HEK293E cells for protein expression; 15 of 22 proteins (68%) were expressed with detection by anti-pentahistidine antibody in our study.

Although the HEK293E cell platform has active N-glycosylation machinery, our candidate list mainly comprised proteins that have yet to be characterized structurally and functionally; out of an abundance of caution, we chose not to modify native sequences in the construct design. We cannot rule out the negative impact of *N*-linked glycans masking critical epitopes, but on this note the literature is variable. Crosnier *et al.* recently evaluated the relative contributions of codon-optimization, an exogenous signal peptide, and mutation of N-glycosylation sites on expression of functional asexual blood-stage protein PfRH5 in the HEK293E cell system, reporting the relative increases in protein expression to be equivocal, ∼3.5-fold, and ∼5.5-fold, respectively, compared with the native sequence (22). By contrast, several other reports exist, albeit in other platforms, wherein retaining native glycans has not made a difference to yield or function (27, 45–48). A recent study from our group using a viral-vectored heterologous prime-boost vaccination regime to deliver Pfs48/45, a protein known to have a complex 6-cysteine domain fold, reports a disruption of transmission-blocking epitopes following mutation of N-glycan sequons relative to the native construct (13). In the present study, glycosylated Pfs25 induced significant levels of TRA, suggesting the chosen construct design and screening strategy can identify a well-known and leading antigen.

Ultimately, seven pre-fertilization and five post-fertilization test candidates were expressed in our platform in enough quantities for evaluation by SMFA. Anti-sera generated to all but one protein (HYP3) bound native parasite protein, but this does not exclude the possibility that antibodies against some critical epitopes are not induced by our vaccine constructs. Pfs47, the only pre-fertilization target selected from the literature screen, is a Pfs48/45 paralog (49) expressed in the female gametocyte and reported to act in parasite evasion of the *A. gambiae* complement system (50). A monoclonal antibody specific to Pfs47 obtained from gametocyte extract vaccination in rats has previously been tested in SFMA, though without TRA in *A. stephensi* mosquitoes (49). Our HEK293-derived Pfs47 did not demonstrate gamete recognition by IFA or any inhibitory effect in *A. stephensi*, but as a member of the 6-cysteine protein family, Pfs47 is known to have a complex fold. As such, we did not pursue SMFA in an *A. gambiae* background. Six additional pre-fertilization candidates (four named and two hypothetical proteins) were tested for TRA from the proteomics screen. Named proteins such as HSP70–2, PDI-8, and PDI-11 were predicted by Wass *et al.* to localize to the *P. berghei* ookinete surface (19). Non-permeabilized ookinetes were detected with both anti-HSP70–2 and anti-PDI-11 sera by IFA in our study, though not in a classical surface distribution, which may reflect some parasite permeabilization during the fixation process. Further imaging would be necessary to confirm the localization, especially as these protein classes are not classically considered to be surface associated.

Post-fertilization targets were identified exclusively from the literature and a systematic knock-out screen (36); SOAP, WARP, PSOP9, PSOP13, and enolase were also expressed in enough quantities for functional assessment. Ookinete micronemal proteins SOAP (secreted ookinete adhesive protein) and WARP (von Willebrand factor A domain-related protein) are implicated, respectively, in the ookinete's initial recognition of (51) and adhesion to the mosquito midgut epithelium during traversal (52). SOAP biology has been elucidated through knockout studies (51), but the candidate has not been previously expressed as heterologous antigen for testing in SMFA. *P. falciparum* recombinant WARP has been expressed in *E. coli*; whole serum tested in SMFA resulted in high TRA (53), which was not replicated with our construct. Neither antigen elicited TRA in SMFA screening in our study. PSOP9 elicited a comparatively high median IgG response by ELISA in cohorts from both Burkina Faso and Mali. Interestingly, PSOP9, also known as GAMA, though never tested in SMFA, has been considered as an erythrocytic-stage vaccine candidate (54); in the same study it was reported to demonstrate strong immunoreactivity among individuals from Thailand and Mali. Apart from the Ecker *et al.* study, PSOP13 has not been heterologously expressed or explored as a TBV immunogen previously; it failed to demonstrate TRA in our study.

Of the test antigens screened, only IgG generated to enolase demonstrated reproducible inhibition of oocyst intensity in SMFA. These findings are consistent with a previous publication by Ghosh *et al.*, which identified enolase as a target of transmission blocking antibodies (55). Although enolase does not have a predicted signal peptide, it is secreted onto the ookinete surface. The antigen was suggested to have a role in midgut recognition and traversal by acting as a hypothetical ligand for a mosquito midgut receptor and means for co-opting mammalian plasminogen, which when converted to active plasmin, aids in midgut traversal. In the Ghosh *et al.* study, enolase was heterologously expressed in *E. coli*. Anti-sera were generated to the recombinant antigen by rabbit immunization; whole sera showed 70% inhibition of *P. falciparum* oocyst intensity by SMFA. In our study, anti-enolase IgG demonstrated moderate though significant inhibitory activity. Unfortunately, low to moderate inhibition is difficult to discern by SMFA (33) unless the same sample is tested in multiple independent assays and/or by a modified SMFA protocol (*e.g.* with dissection of many more mosquitoes). Indeed, the other two IgG samples of interest (anti-HSP70–2 and anti-HYP1) demonstrated variable levels of inhibition in independent feeds. Further feeding experiments would be required to determine whether anti-HSP70–2 or anti-HYP1 IgG have true, but weak, inhibitory activities biologically relevant to the *P. falciparum* transmission pathway.

In totality, HEK293-based recombinant protein expression and SMFA screening represents a robust, moderate-throughput approach for capitalizing on rapidly growing protein expression data for the sexual stages. Screening presents an unbiased approach toward elucidating key components of the transmission pathway and re-invigorating the pre-clinical TBV pipeline. In keeping with published literature (13, 14), our data support a strong inhibition of oocyst intensity by anti-Pfs25 IgG; at present, Pfs25 remains the best-characterized TBV antigen. Given enolase's moderate inhibition, to be of value for TBV development, this antigen would need to enhance functional antibody response generated to Pfs25, and the production and evaluation of chimeric protein may thus be an interesting future experiment.

## Supplementary Material

Supplemental Tables

## Footnotes

^1^ The abbreviations used are:

VIMT: vaccines interrupting malaria transmission
TBV: transmission-blocking vaccines
SMFA: standard membrane feeding assay
IFA: indirect fluorescence assay.
