# Supplementary material for: Functional Characterization and Comparison of Plasmodium falciparum Proteins as Targets of Transmission-blocking Antibodies
Source: Mol Cell Proteomics. 2017 Oct 31;19(1):155–66. doi: 10.1074/mcp.RA117.000036 (PMC6944241; doi:10.1074/mcp.RA117.000036)
Supplement: Supplemental Tables [file 4579_1_supp_7636_twvjvx.pdf]

Supplemental Table 1 Total candidate list from unbiased proteomics review

Accession	Gene Name	Size	TMD	Troph/Schiz	Gametocyte	Gamete	Total
PF3D7_1222300	HSP90	95	0	88	126	134	348
**PF3D7_0917900**	HSP70-2, BIP	72.4	0	151	122	135	408
PF3D7_1324900	LDH	34.1	1	29	61	64	154
PF3D7_0406200	Pfs16	16.6	2	5	58	47	110
PF3D7_1346700	Pfs48/45	51.5	2	3	56	27	86
**PF3D7_0827900**	PDI-8	55.5	1	76	55	80	211
PF3D7_1108600	ERC	39.4	0	49	33	42	124
PF3D7_0721700	PSOP1	53.3	0	0	26	18	44
PF3D7_1104400		49.3	1	25	20	18	63
**PF3D7_0912400**		52.7	1	3	19	5	27
PF3D7_1031000	Pfs25	24.1	1	0	18	7	25
**PF3D7_1449000**	GEST	28.8	0	0	18	9	27
PF3D7_1352500		24	2	33	16	11	60
**PF3D7_0925900**		24.7	0	10	13	14	37
PF3D7_1115600	CYP19B	21.7	0	11	13	8	32
PF3D7_1132800	AQP/AQP1	28.3	6	8	10	5	23
PF3D7_0509600	AsnRS	85.3	1	1	8	5	14
PF3D7_1331800		15	0	9	7	1	17
PF3D7_1232100	CPN60	81.5	1	10	5	2	17
PF3D7_1471100	EXP2	33.4	0	29	5	2	36
**PF3D7_0303900**		23.2	0	0	4	7	11
PF3D7_1319800		16.8	1	0	4	2	6
PF3D7_0629200		44.7	1	6	3	5	14
PF3D7_1129100	PV1	52	0	11	3	4	18
PF3D7_0113900	CBP1, GEXP10	28.2	3	2	2	2	6
PF3D7_0513000		31	0	0	2	1	3
PF3D7_0828800	PSOP9, GAMA	85.3	2	0	2	1	3
**PF3D7_1134100**	PDI-11	49.2	0	0	2	2	4
**PF3D7_0911900**	ICP	47	0	0	1	2	3
PF3D7_1010700		25.7	0	2	1	1	4
PF3D7_1108700	Pfj2	62.4	1	2	1	1	4

Accession numbers in bold represent candidates which were selected for expression. Size is reported in kiloDaltons. Abbreviations: TMD: number of predicted transmembrane domains; Columns denoted Troph/Schiz (throphozoite/schizont), Gametocyte, Gamete, and Total represent the number of stage-specific and total peptides identified by the Lasonder *et al* proteomics screen.

Supplemental Table 2 Percent inhibition of mean oocyst intensity by purified IgG generated to *P. falciparum* proteins in SMFA – Repetition 1

Data for SMFA repetition 1 was collected over two independent feeding experiments, denoted A and B below; anti-OVA and anti-Pfs25 controls were included in each independent feed.

Sample	IgG (mg/mL)	Mean Oocyst	% Oocyst Inhibition	95% CI Low	95% CI High	p-value
Independent feeding experiment A						
OVA	0.750	38.5				
Pfs25	0.750	0.0	100.0	99.7	100.0	0.001
PSOP13	0.750	34.8	9.7	-160.7	68.4	0.910
Enolase	0.750	20.6	46.6	-50.8	81.3	0.225
PDI-8	0.750	34.1	11.6	-164.7	69.9	0.816
HYP1	0.750	20.9	45.7	-56.4	81.9	0.259
HYP2	0.750	35.9	6.8	-164.9	70.2	0.927
Independent feeding experiment B						
OVA	0.750	29.5				
Pfs25	0.750	0.1	99.8	98.7	100.0	0.001
SOAP	0.750	38.7	-31.4	-283.1	53.5	0.580
WARP	0.750	27.4	7.0	-170.6	68.6	0.907
HSP70-2	0.750	35.4	-20.0	-227.8	58.8	0.750
PDI-11	0.750	34.7	-17.7	-254.4	59.2	0.762
ICP	0.750	42.2	-43.3	-321.3	47.6	0.499
Pfs47	0.750	33.5	-13.6	-228.1	62.0	0.797
PSOP9	0.750	33.7	-14.4	-232.8	60.9	0.781

Supplemental Table 3 Percent inhibition of mean oocyst intensity by purified IgG generated to *P. falciparum* proteins in SMFA – Repetition 2

Data for SMFA repetition 2 was collected in a single independent feeding experiment.

Sample	IgG (mg/mL)	Mean Oocyst	% Oocyst Inhibition	95% CI Low	95% CI High	p-value
OVA	0.750	39.9				
Pfs25	0.750	0.1	99.9	99.1	100.0	0.001
PSOP13	0.750	38.1	4.5	-174.7	66.6	0.953
Enolase	0.750	18.8	52.9	-40.7	84.2	0.179
PDI-8	0.750	32.1	19.5	-137.8	70.5	0.727
HYP1	0.750	26.4	33.8	-101.6	77.1	0.448
HYP2	0.750	49.8	-24.8	-271.7	55.9	0.698
SOAP	0.750	36.4	8.8	-165.2	68.9	0.863
WARP	0.750	27.4	31.5	-109.4	76.9	0.481
HSP70-2	0.750	0.7	98.2	94.5	99.7	0.001
PDI-11	0.750	36.6	8.4	-171.2	69.6	0.880
ICP	0.750	35.1	12.2	-152.8	69.3	0.784
Pfs47	0.750	35.4	11.4	-153.4	68.9	0.811
PSOP9	0.750	35.9	10.2	-157.4	69.8	0.855
